# Greenness and equity: Complex connections between intra-neighborhood contexts and residential tree planting implementation

**DOI:** 10.1016/j.envint.2023.107955

**Published:** 2023-05-12

**Authors:** Ray Yeager, Matthew H.E.M. Browning, Elizabeth Breyer, Alessandro Ossola, Lincoln R. Larson, Daniel W. Riggs, Alessandro Rigolon, Christopher Chandler, Daniel Fleischer, Rachel Keith, Kandi Walker, Joy L. Hart, Ted Smith, Aruni Bhatnagar

**Affiliations:** a Christina Lee Brown Envirome Institute, University of Louisville. 302 E Muhammad Ali Blvd, Louisville, KY 40202, USA; b Division of Environmental Medicine, Department of Medicine, University of Louisville, 302 E Muhammad Ali Blvd, Louisville, KY 40202, USA; c Superfund Research Center, University of Louisville, 302 E Muhammad Ali Blvd, Louisville, KY 40202, USA; d Center for Integrative Environmental Health Sciences, University of Louisville, 302 E Muhammad Ali Blvd, Louisville, KY 40202, USA; e Department of Parks, Recreation, and Tourism Management, Clemson University, Sirrine 120B, Clemson, SC, USA; f Texas A&M University, Department of Geography. Building 0443, 797 Lamar St, College Station, TX 77843, USA; g Department of Plant Sciences, University of California Davis. PES-1238, One Shields Avenue, Davis, CA 95616, USA; h College of Natural Resources, North Carolina State University. Biltmore Hall 4008L, Raleigh, NC 27695, USA; i Department of City and Metropolitan Planning, The University of Utah. 375 S 1530 E, RM 204 ARCH, Salt Lake City, UT, 84112, USA; j North American Cities Network, The Nature Conservancy. 308 Central Ave, Pewee Valley, KY 40056, USA; k Hyphae Design Laboratory, 942 Clay Street, Oakland, CA 94607, USA; l Department of Communication, University of Louisville, 2301 South 3rd Street, Louisville, KY 40292, USA

**Keywords:** Greenness, Socioeconomic Status, Environmental Justice, Sustainability, Planting, Implementation

## Abstract

Associations between neighborhood greenness and socioeconomic status (SES) are established, yet intra-neighborhood context and SES-related barriers to tree planting remain unclear. Large-scale tree planting implementation efforts are increasingly common and can improve human health, strengthen climate adaptation, and ameliorate environmental inequities. Yet, these efforts may be ineffective without in-depth understanding of local SES inequities and barriers to residential planting.

We recruited 636 residents within and surrounding the Oakdale Neighborhood of Louisville, Kentucky, USA, and evaluated associations of individual and neighborhood-level sociodemographic indicators with greenness levels at multiple scales. We offered no-cost residential tree planting and maintenance to residents within a subsection of the neighborhood and examined associations of these sociodemographic indicators plus baseline greenness levels with tree planting adoption among 215 eligible participants.

We observed positive associations of income with Normalized Difference Vegetation Index (NDVI) and leaf area index (LAI) within all radii around homes, and within yards of residents, that varied in strength. There were stronger associations of income with NDVI in front yards but LAI in back yards. Among Participants of Color, associations between income and NDVI were stronger than with Whites and exhibited no association with LAI. Tree planting uptake was not associated with income, education, race, nor employment status, but was positively associated with lot size, home value, lower population density, and area greenness.

Our findings reveal significant complexity of intra-neighborhood associations between SES and greenness that could help shape future research and equitable greening implementation. Results show that previously documented links between SES and greenspace at large scales extend to residents’ yards, highlighting opportunities to redress greenness inequities on private property. Our analysis found that uptake of no-cost residential planting and maintenance was nearly equal across SES groups but did not redress greenness inequity. To inform equitable greening, further research is needed to evaluate culture, norms, perceptions, and values affecting tree planting acceptance among low-SES residents.

## 1. Introduction

Tree and vegetation planting on homeowner’s lots can improve public health, enhance climate adaptation, and ameliorate the burden of environmental inequities (James et al., 2015; Nesbitt et al., 2018; Tubridy, 2020; Chiabai et al., 2018; Iungman et al., 2023). Such greenness benefits health and sustainability by mitigating urban heat, reducing stormwater runoff, lowering air pollution, absorbing noise pollution, improving mental health, enabling recreation and exercise, and facilitating social interaction (James et al., 2015; Davis et al., 2016; Zabret and Sraj, 2019; Larson et al., 2022; Villeneuve et al., 2018; Wan et al., 2021; Remme et al., 2021). However, greenness is often concentrated among higher-income and education communities and racial-majority populations, depriving more marginalized populations the benefits of greenery (Klompmaker et al., 2023; Rigolon, 2016; Wolch et al., 2014; Nesbitt et al., 2019; Schwarz et al., 2015).

Despite observations from many prior studies on the links and mechanisms of connection between socioeconomic status (SES) and greenness, few studies have directly investigated intra-neighborhood and individual-level indicators of SES and proximate greenness. Most previous studies of inequities in residential greenness evaluate participant cohorts or neighborhoods that are widely geographically dispersed (De Vries et al., 2020; Sathyakumar et al., 2019; Allegretto et al., 2022; Shackleton et al., 2014; Hernández and Villaseñor, 2018; Nero et al., 2018). Few detailed evaluations have examined how proximate greenness of residents’ yards and nearby areas relate to sociodemographic attributes among individuals within a neighborhood. A greater understanding of these spatially-layered factors is critically important to evaluate and address inequities in greenness. Further needed is context of the specific attributes of greenness that may be particularly inequitable, such as the volume of tree canopy as well as comprehensive measures (i.e., total vegetative cover). Importantly, different greenness attributes and spatial arrangements may correspond to mediators and moderators through which greenness affects health outcomes, subsequently affecting health inequities that may vary based on the spatial extent of vegetation assessed (Browning and Lee, 2017; Yeager et al., 2020). For example, mental health may be supported from nearby greenery, such as window views of front and back yards (Labib et al., 2020; Zhang et al., 2023). Meanwhile, physical activity and cardiovascular outcomes may benefit from increased walkability and air quality from greenery across entire neighborhoods (Browning and Lee, 2017; Yeager et al., 2020).

Importantly, there is little previous research on the extent to which offers of no-cost tree planting and maintenance on private lots are accepted at higher rates by residents with lower SES and less baseline greenness. A previous evaluation of free and reduced-cost planting programs in the U.S. cities of Washington D.C. and Baltimore, Maryland found both higher existing canopy and higher levels of planting among affluent neighborhoods but was unable to examine individual plantings within neighborhoods (Locke and Grove, 2016). This study reported that existing outreach strategies to plant free and reduced-cost trees were “extraordinarily effective” in affluent communities and substantially less effective in less affluent communities. Another study conducted in the East side of the U.S city of Portland, Oregon found that individuals with greener home lots and within higher education census block groups were more likely to accept a no-cost tree planting (Donovan and Mills, 2014). Both studies highlight the potential for unequal and inequitable common approaches for engagement and planting to improve urban canopy but could not evaluate associations between individual-level SES and planting success.

Many efforts are underway in the U.S. and worldwide to improve access to healthy and safe green space in low-SES communities needing conducive environments and climate solutions to support the health and well-being of their citizens (Chiabai et al., 2018; Eldridge et al., 2019; Yañez et al., 2021; Oscilowicz et al., 2021). Nature-based investments can be made in parks, school yards, rights-of-way, and other public areas. However, the majority of plantable area in and around where most people spend the vast majority of their time (e.g., residential neighborhoods) is on private residential lots (Ossola et al., 2018). Therefore, an understanding individual-scale greenness and acceptance of planting on their property in areas of need is essential when attempting to address environmental inequities across larger scales. To address such gaps in knowledge, we conducted an evaluation of a large-scale planting campaign, a component of the Green Heart Louisville greenness and health study, to examine associations of individual-level SES indicators with greenness and residential tree planting adoption. For this evaluation, we sought to build on prior evidence describing links between greenness and SES by evaluating two main objectives: 1. determine the extent to which intra-neighborhood associations between greenness and SES are dependent on contextual factors of spatial scale, greenness attributes, and individual characteristics; and 2. evaluate whether a well-advertised and no-cost tree planting campaign was successful in overcoming socioeconomic barriers to equitable planting.

## 2. Materials and methods

### 2.1. Study area and participants

This study is part of the larger Green Heart Louisville Project. This project involves a longitudinal cardiovascular risk cohort in Louisville, Kentucky, USA. As part of this study, we recruited 733 participants at baseline from the urban Oakdale residential neighborhood and surrounding residential areas in the summers of 2018 and 2019. The 2017–2021 American Community Survey median annual household income of census tracts in the study area ranged from 23,632 to 49,608 USD, compared with a median 61,633 USD across Louisville-Jefferson County (United States Census Bureau and Survey, 2021; Living Atlas, 2023). The study area is 13 km^2^, with 41.54% single-family residential housing area, 2.38% apartment area, 2.15% other multifamily housing, and 4.4% commercial horse racing facilities. Other property types are not well characterized by municipal cadastral records but mainly consist of roadway and right-of-way areas. Limited areas of commercial, industrial, park, and vacant land are also present.

We recruited participants during the study baseline enrollment period via community events, mailers, geotargeted online advertisements, social media posts, and door-to-door recruitment and flyers. All study procedures were reviewed and approved by the University of Louisville Institutional Review Board (IRB #15.126). All participants provided informed consent before data were collected. Participants were invited to an in-person exam and survey administration at 1 of 5 potential study sites within the study area. Of 733 enrolled study participants, 97 did not meet inclusion criteria for the current analysis due to incomplete necessary data or an address that could not be located; thus, they were not considered. All individual-level data analyzed were collected from the survey administration at the time of enrollment.

### 2.2. Data collected

#### 2.2.1. Sociodemographic attributes

We collected individual-level data on SES and other demographic characteristics via questionnaires at the time of the study enrollment: categorical ranges of current household income, educational attainment, maternal educational attainment, and employment status (Duncan et al., 2002; McDermott, 2018; Lantz and Pritchard, 2010). We geocoded the reported home addresses of participants to generate additional individual-level data. We attributed property value from municipal records based on reported participant addresses (Jefferson County Property Valuation Administrator Office, 2018).

#### 2.2.2. Participant demographics and area characteristics

We collected other participant demographics from a questionnaire at the time of enrollment: age, gender, race, ethnicity, and home address. From geocoded participant records, we extracted overlapping data: lot size, roadway traffic, and population density. We collected residential parcel lot size from municipal records. (Jefferson County Property Valuation Administrator Office, 2018) We assigned nearby population density data to participants based on block groups data from the U.S. Census Bureau American Community Survey 2017–2021 5-year estimates (United States Census Bureau and Survey, 2021). No other area-level population data were employed due to missing data for some block groups in the study area and substantial bias and misclassification that would result from applying tract-level data to the spatially confined and clustered participant cohort. As a proxy for substantially different landcover from residential areas as well as air and noise pollution, we calculated road traffic density on major roads as the number of vehicles passing per day within 500 m of the home using daily traffic count data for major roadway segments, provided by the Kentucky Transportation Cabinet.

#### 2.2.3. Greenness measurements

We used two metrics to assess greenness: Normalized Difference Vegetation Index (NDVI) and Leaf Area Index (LAI). NDVI is a commonly employed remotely sensed metric in greenness and health studies due to its widespread availability and representativeness of landcover by photosynthetically active vegetation. Most NDVI measurements for individual-level evaluations are limited by coarse spatial resolution. Canopy data can be used to overcome this limitation but is itself limited to two-dimensional cover estimates and not three-dimensional values (i.e., total volume). To address these limitations of greenness metrics evaluated in previous studies, we calculated high-resolution measures of both NDVI and LAI to represent total vegetation cover (NDVI) and the total leaf area of trees (LAI), which is more representative of the total tree volume per unit of land area and the ecosystem services provided by trees than canopy cover measurements alone. We compiled NDVI and LAI on each participants’ lot, including their front and back yards, and Euclidean spatial buffers of 20 m, 50 m, 100 m, 150 m, 200 m, 250 m, 300 m, 400 m, and 500 m. Front and back yards were geolocated following a supervised algorithm that splits each residential parcel into two based on the reciprocal position of the front street centerline and the main building (I.e., house) centroid.

The high-resolution imagery used for NDVI and LAI metrics was collected using light detection and ranging (LiDAR) data and multi-spectral imagery at 20 points per meter resolution from a commissioned fixed-wing aircraft, in other words an average of 20 laser return points per square meter of land area were collected and utilized to create a 3-dimensional point cloud in GIS software. This aircraft conducted flyovers of the study area on cloud-free days, August 17th-19th, 2019. LAI raster surface was extracted from the aerial LiDAR data using a method based on the Beer-Lambert law (Klingberg et al., 2017). Multispectral imagery used to calculate NDVI was retrieved from Planetscope DOVE satellites at 4 m^2^ resolution during cloud-free days in 2019. To account for intra-season vegetation phenology, we calculated a summer NDVI mean raster surface from seven individual time points, spaced at minimum 10 days apart, in the summer of 2019 during the months of May (1), June(2), July(1), August(1), and September(2).

We calculated mean NDVI and LAI values using the Focal Statistics tool in ArcGISPro version 3.x (ESRI, Redlands, CA). We utilized raster-based NDVI and LAI greenness values at original resolution of the input data. This process yields a continuous raster surface where each raster cell represents the mean value of all cells within 100 m on the input raster surface. We then extracted individual participant greenness values from this focal statistics raster output surface, representing average greenness of each radius, at the geocoded point of participant residences. For spatial units of front yards and back yards, we utilized the ArcGISPro Neighborhood Statistics tool to calculate mean NDVI and LAI for each study participant, with front and back yard spatial polygons and original greenness raster data as the input.

#### 2.2.4. Tree planting adoption

The Green Heart Louisville Project separated the study area into a target planting area and a matched surrounding control area with no dedicated plantings. We conducted extensive advertisement and recruitment efforts to recruit individuals in the target planting area to accept a no-cost tree planting and ongoing maintenance (i.e., watering, pruning, and replacement if necessary) for two-years either (a) on their property at their residence or (b) on associated right-of-way areas between their residential parcel and the street. Recruitment and advertisement were conducted by project staff at 148 community events with approximately 5,000 total attendees and via multiple rounds of mailers to all eligible residences, postings on social media, and in-person recruitment from door-to-door knocking and flyers. Study participants were made aware of planting efforts during Green Heart study visits and further contacted to ensure awareness of planting eligibility. Recruitment and plantings were conducted from April 2019 to June 2022, with intermittent interruptions due to unplantable seasons, unsuitable weather, and the COVID-19 pandemic. We collected geographic positioning system (GPS) coordinates for each successfully planted tree after planting. Due to GPS logging accuracy and right-of-way areas not classified as part of home lots, we assessed a participant residence as being planted (i.e., success tree adoption) if a tree planting was recorded on or within 5 m of each participant’s residential parcel.

### 2.3. Statistical analyses

All statistical analyses were conducted in RStudio version 2022.12.0 software. To evaluate differences in participant characteristics between levels of greenness, we categorized NDVI into tertiles (low, medium, high) within a 100 m radius. We represented participant characteristics as n (%) for categorical variables and mean (SD) for continuous variables.

We utilized generalized linear models to estimate associations between individual-level SES indicators and both NDVI and LAI in front yards, back yards, and with multiple spatial radii around residential addresses. We grouped categorical SES measures into secondary categories with either a comparable number of participants, or high versus low as appropriate, with the lowest SES category as the reference group. Effect size estimates represents estimated difference in the mean greenness value of each participant variable category when compared with the reference group. We tested associations between SES and greenness at spatial scales of front yards, back yards, and spatial radii of 20 m, 50 m, 100 m, 150 m, 200 m, 250 m, 300 m, 400 m, and 500 m. To assess associations between SES and greenness independent of potentially confounding factors, we adjusted regression models for participant age, gender, race, income, education, mother’s education, employment status, and traffic density.

In a series of stratified analyses to examine potential differences in associations between greenness and SES among distinct groupings of participants, we used the same model covariates to test for associations within select participant groups (i.e., younger vs. older) while excluding covariates where models were stratified by the same variable. Groupings included White and People of Color; those at or over age 62 (retirement age defined as those eligible for social security) and those under 62; those with a college degree or higher and those without; traffic exposure within 500 m split evenly between the higher and lower exposure participants; NDVI mean within 500 m split evenly between higher and lower; and LAI mean within 500 m split evenly between higher and lower.

To evaluate success of planting, we compared characteristics of participants that accepted residential planting with those who did not using ANOVA or chi-squared tests as appropriate. We utilized ANOVA and chi-squared approaches to assess significant differences in participant and area characteristics between eligible participants residing in a single-family home whose residence was planted and those not planted. We included all participant measures of SES, gender, age, population density, traffic density, assessed home value, lot size, and both metrics of greenness at distinct spatial scales of yards, 100 m radius, and 500 m radius.

## 3. Results

### 3.1. Overview of observations

**Fig. 1.**
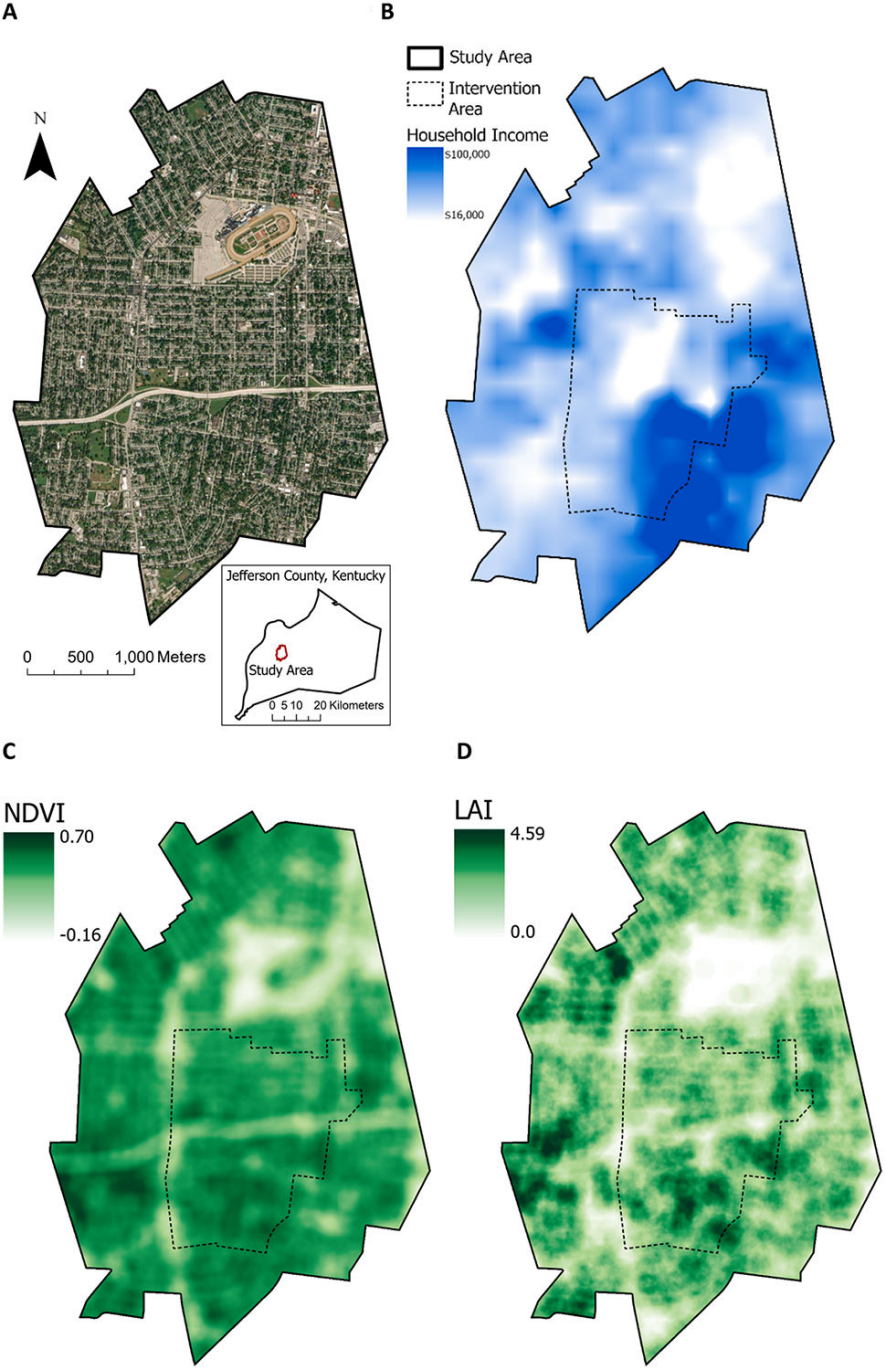
Demographic attributes and greenness across the study area. A, True-color image and county inset map of the study area. B, Income of participants, displayed as deidentified location-masked interpolated mean of categorical income ranges. C, NDVI within 100 m distribution across the study area, displayed by standard deviation stretched scale. D, LAI within 100 m distribution across the study area, displayed by standard deviation stretched scale.

We observed substantial geographic variability in income and greenness across the study area, with landcover type, primarily consisting of residential, transportation, and private business coverage, driving the starkest differences in greenness (Fig. 1). Participants’ incomes were generally higher in the southeast and lower in the northeast. NDVI was relatively high throughout the study area except for the northeast and along major roadways. LAI was higher in the southwest and southeast. Localized variations in greenness were largely driven by business areas, parking lots, roadway corridors, and a large horse racing complex in the north-central portion of the study area. Due to minor differences in sensor collection bandwidth, our calculated NDVI values were lower than those that would be found in commonly used NDVI calculations based on data from Landsat satellites.

### 3.2. Unadjusted associations between greenness and SES

We observed significant associations between higher tertiles of NDVI within 100 m and higher income levels, higher shares of single-family home residences, higher home value, lower traffic density, higher census tract level income, and lower census tract level population density (Table 1). No other individual- or neighborhood-level characteristics were associated with NDVI, *p* > 0.05.

### 3.3. Adjusted associations between greenness and SES

Linear regression models (Table 2) showed that higher-income individuals had higher average NDVI values in their front and back yards and within 20 m to 500 m of their home. We observed similar associations for participants in the middle-income group, except the effect sizes were smaller and not all associations were significant. Few associations with other SES indicators were significant, and the direction of associations were mixed. For instance, lower levels of maternal education were associated with lower average NDVI values in 50–100 m radii, while being employed was associated with lower NDVI values in the front yard.

We also observed higher average LAI levels among middle- and higher-income participants. Positive associations between LAI and the high-income group were significant among all buffer sizes and marginally significant among back yards. Positive associations were also significant between LAI and the middle-income group for a 500 m radius and marginally significant for front and back yards, as well as 20 m and 400 m buffers. The strongest effects were once again seen for the residential yards and at smaller buffer sizes, but were marginally significant, with the largest effect seen when comparing high vs. low income back yard LAI. We observed few significant associations between greenness metrics and indicators of SES other than income. For education, we found a marginally significant association between those with a college degree and higher NDVI in a 500 m radius, as well as significant associations with LAI at 400 m and 500 m radii. We also found that participants with college-level educational attainment had a marginally significant association with lower back yard LAI than the referent group. For maternal education, we observed significant associations with some college education and lower NDVI at 50 m and 100 m radii, but higher LAI in back yards. Employment status was only significantly associated with front yard NDVI.

### 3.4. Stratified analysis

**Fig. 2.**
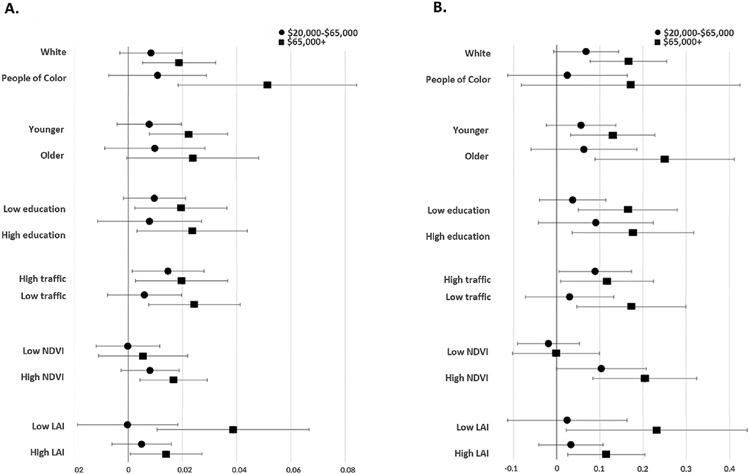
Stratified analyses between income and NDVI (A) and LAI (B) in 100 m around the home, stratified by participant characteristics. Notes: N = 636. Effect estimates of linear regression models adjusted for participants’ age, gender, race, income, education, mother’s education, employment status, and traffic density with 95% confidence intervals (CI) of the middle (circles) and high (squares) income groups. Reference group was the low-income group (annual household income < 20,000 USD). A. 100 m NDVI and Income, B. 100 m LAI and income. NDVI and LAI stratifications represent associations between income and greenness within 100 m stratified by high and low greenness values within a 500 m radius.

Stratified analyses also found positive associations between income and greenness within select demographic groups (Fig. 2). The highest income group, but not the middle-income group, and higher NDVI, with Participants of Color displaying a much larger effect size than White participants. While we also observed significant positive associations between income and LAI among White participants, we did not observe a similar association between LAI and income among Black participants. When stratifying participants by those of retirement age (age 62 + ), we observed significant positive associations between both NDVI and LAI with high income among younger participants, but only LAI among older participants. For participants with both high and low educational attainment, we observed significant positive associations between the highest income group and both metrics of greenness, but not the middle-income group. We observed significant positive associations between both high and medium income and both metrics of greenness among those with low proximate traffic density. However, for participants with higher proximate traffic, only those with high income were significantly positively associated with NDVI and LAI. When comparing associations of income and greenness within a 100 m radius between those with the higher and lower greenness within a 500 m radius, we only found significant positive associations between greenness and income among those with living in already high greenness areas. We found significant positive associations between the highest income participants and greenness among participants living in both single- and multi-family housing structures. However, the effect size of this association was substantially larger among those living in single-family residential structures.

### 3.5. Adoption of no-cost tree plantings

Of the 215 study participants living within a single-family residential structure and eligible for no-cost planting and maintenance, 95 chose to accept a tree planting. Unlike for greenness, we observed no evidence that participants’ sociodemographic characteristics were related to tree planting adoption (Table 3). However, planting was more likely for residents with larger lot sizes and higher home values and those living in areas with a lower population density. Furthermore, planting adoption was more likely for participants with higher baseline greenness measures, more so LAI than NDVI. Significant factors were restricted to NDVI and LAI measured in 100 and 500 m buffers and excluded any front or back yard greenness estimates.

## 4. Discussion

### 4.1. Interpretation and context of findings

We found significant positive individual-level associations between income and greenness, similar to observations of previous investigations, but no consistent associations with other measures of SES. However, we observed heterogeneity of these associations with participant characteristics, greenness indicators, and especially spatial extent of greenness assessment. Eligible participants with low income were not more likely to accept no-cost residential tree plantings than participants with higher incomes, indicating that the removal of the financial barriers of planting and maintenance may not alleviate inequalities in greenness among low-SES populations.

The pattern of areas of lower income having lower greenness, observed many times before, was repeated when we examined associations between greenness and income at the individual parcel-level. However, when we examined adjusted associations between greenness and indicators of SES among distinct metrics of greenness and numerous spatial scales, substantial nuances of this relationship became evident. Effect sizes of the association between the highest income group compared to the reference low-income group and greenness were more than double at some spatial scales compared with others for NDVI, and more than triple for LAI. Income was substantially more associated with front yard NDVI than back yard or any radii assessed, and a similar effect of LAI in back yards was found but not in front yards. While reasons for these differences between metrics and between front and back yards are unknown, it is clear that there are strong associations between income and greenness at individual residences. This finding indicates that low-income participants are not only subjected to lower greenness in residential areas, but lower greenness at their individual residences, potentially impairing the benefits of highly proximate greenness such as heat mitigation, household recreation, and mental health benefits from views of nature. Of radii around homes, we observed the largest effect size between income and greenness at a 100 m radius for both NDVI and LAI. At scales between 150 m and 500 m there were lower but generally consistent effect sizes. Unlike participant income, we observed no consistent associations between other SES indicators and greenness at multiple spatial scales. Although these results cannot be extrapolated to other cohorts, they indicate that prior epidemiological investigations linking greenness with health outcomes may overlook important spatial context at the local parcel-based scale.

Associations between income and greenness at 100 m were generally consistent between participants with different educational attainment levels, nearby traffic, 500 m radius greenness, and house type, but differed with age and race. While associations between income and greenness among White participants were similar to overall observations, income was only significantly associated with NDVI, and not LAI, among Participants of Color with a substantially larger effect size than any other participant strata. This observation could potentially reflect inequitable historic planting and maintenance policies or substantial barriers to planting and maintenance beyond plantable area and economic ability. Associations between income and greenness among participants below retirement age mirrored the overall cohort but were only significant for LAI among those of retirement age without concurrently elevated NDVI. We observed a similar pattern of associations as the overall cohort for both residents of single- and multiple-family structures, but substantially larger effect sizes among residents of single-family structures. This finding likely reflects a more direct relationship between income and greenness among areas with more direct agency over residential greenness and generally lower proximity to businesses and roadways with fixed greenness levels (Zhou et al., 2009).

Differences in associations between front and back yards may reflect understandings of back yards as private spaces where residents customize environments to personal preferences, whereas front yard attributes may be more influenced by adherence to perceived social norms (Ossola et al., 2019; Locke et al., 2018). While neighborhoods would likely benefit from greening in nearly any plantable location, front yard greening could confer more collective benefits of walkability, perceived neighborhood quality, and mitigation of roadway pollution. However, more research is needed to elucidate the potentially disparate effects of front or back yard greenness on instauration and restoration pathways to affect health.

Taken together, we found clear and consistent positive associations between income and greenness at the individual level, which supports the idea that financial constraints to planting and maintenance are a major driver of inequitable levels of greenness among low-income participants (Watkins et al., 2017). If true, we might expect high levels of planting uptake among low SES individuals resulting from a well-advertised residential planting campaign offering trees, installation, and two years of maintenance at no cost to residents. However, this hypothesis was not consistent with our observations of planting among the 215 study participants who were eligible. In contrast, we observed a significant positive association between one SES indicator and planting, with more planting success among those with higher home values, potentially driven by lot size. Consistent with this observation of lower planting success among those with smaller lots, we also found less success in areas with a higher population density. Unexpectedly, higher area-level greenness, mainly LAI, was also significantly associated with more planting success. While reasons for this finding are unclear, we hypothesize that for those living in more foliated areas, residential trees may be more normalized and residents may have a more informed perception of the costs versus the benefits of trees (Schwarz et al., 2015; Vogt et al., 2015). While not directly comparable with findings based on area-level measurements of SES conducted across larger study areas, we observed a similar trend of greater planting success in greener areas, but unlike those studies, very limited positive associations between uptake and SES (Allegretto et al., 2022; Locke and Grove, 2016; Donovan and Mills, 2014). Notably, as we utilized data provided by Green Heart health study participants, with study branding and engagement directly connected to planting recruitment activity, all eligible participants were made aware of the planting opportunity. Yet, awareness among those eligible was still not enough to overcome planting inequities that have been observed elsewhere, implying barriers to planting among low SES communities that go well beyond unequal advertising success.

Past research has revealed a number of reasons why tree planting efforts in low-income neighborhoods might be met with unexpected resistance, including lack of awareness of potential benefits, financial burdens, perceived gentrification risk, stewardship concerns, insufficient solicitation of civic involvement, and trust and relationship barriers (Donovan and Mills, 2014; Riedman et al., 2022; Carmichael and McDonough, 2019; Breyer and Mohr, 2023). Regardless of the underlying reasons, our well-advertised, community-engaged, and no-cost tree planting and maintenance campaign had near-equal, but still inequitable, success across socioeconomic groups and was not sufficient to overcome socioeconomic disparities in residential greenness. Reports from community engagement and interactions suggest a wide number of individual-specific concerns that might impact tree uptake, often centered on topics of maintenance, perceptions of enough nearby trees already, or concerns about the suitability of yards for trees.

### 4.2. Strengths and limitations

Our study had several important limitations. The Green Heart Louisville Project cohort was restricted to a single area of an eastern U.S. city. Therefore, our results may not generalize to other areas of the city or other cities across the country. Still, the cohort’s demographics were similar to many lower- to moderate- SES urban communities throughout the U.S. Furthermore, our participant cohort likely suffers from some selection bias of those more willing and able to enroll in a clinical study with biological measurements, specimen collection, and questionnaire completion. While the relatively small study area enabled high-spatial resolution greenness using measures such as LiDAR collection, such measures may not be feasible for larger areas; therefore, our estimates may differ from other studies’ results using less precise estimates. Additionally, we could not utilize more detailed measures of SES such as wealth, vulnerability, economic stability, social capital, or perceived social class, as this information was included in the study questionnaires designed to evaluate links between greenness, pollution, and cardiovascular health. Finally, we identified single-family residential structures and property values from municipal property records, but we do not have data identifying whether the participants’ owned or rented these properties. Furthermore, although we documented disparities in tree uptake across neighborhood context, more research is needed to understand why these disparities persist (Riedman et al., 2022).

The study also had several strengths. We included individual-level data with spatially precise metrics of greenness, enabling the evaluation of typically unattainable spatial scales of radii less than 100 m and front versus back yards. We also assessed LAI as a more detailed measure of the presence of trees than percent canopy cover, which misrepresents tree height and volume. Another notable strength is the large-scale greenness intervention nested within the study area. This intervention utilized an unprecedented level of community engagement, outreach, and recruitment of residents to develop awareness and provide no-cost planting and maintenance on private residential property. Given the lengths taken to facilitate planting uptake, it is possible that similar efforts without such extensive engagement activities may be less successful in reaching low-SES residents.

### 4.3. Conclusions

The present analysis provides clear evidence of variations in links between existing greenness and SES by attributes of greenness, spatial scale, and individual characteristics within one neighborhood area in an eastern U. S. city. We found no evidence that residents with less greenery around their home or lower SES were more likely to accept a no-cost tree planting and two-years of maintenance, thus demonstrating equal but inequitable planting success. These results indicate a need for future planting evaluation that could help to inform and tailor implementation approaches to best achieve equitable greening practices (Riedman et al., 2022; Carmichael and McDonough, 2019). Although such efforts may increase the overall costs of tree planting, they are important to impart the large and sustainable financial and ethical return on investment through the resulting health benefits and ecosystem services provided by greener environments among communities of historic disadvantage and greatest present need (Remme et al., 2021; Chiabai et al., 2018; Iungman et al., 2023; Davis et al., 2016; Van Den Eeden et al., 2022; Browning and Rigolon, 2019; Nigussie et al., 2021; Becker and Browning, 2021; Brown et al., 2016; Spahr et al., 2020; Xi et al., 2023; Nowak and Aevermann, 2019; Roman et al., 2015). Thoughtful and informed approaches to address greenness needs and barriers to tree planting beyond affordability are critical to multiply the effectiveness of similar efforts in the future.

## Figures and Tables

**Table 1:** Individual- and neighborhood-level characteristics of participants and unadjusted associations with NDVI (N = 636). Notes: Chi-Sq and ANOVA associations by low (0.06–0.19), medium (0.19–0.23), and high (0.23–0.36) tertiles of NDVI within a 100 m radius. Area population density defined at the block group level.

		NDVI, 100 m Radius			
Categorical – n (%)	Total, N = 636	Low, N = 212	Medium, N = 212	High, N = 212	p-value
**Gender**					0.592
Female	391	136 (64.2)	129 (60.8)	126 (59.4)	
Male	245	76 (35.8)	83 (39.2)	86 (40.6)	
**Race**					0.191
Black	104	44 (20.8)	30 (14.2)	30 (14.2)	
White	497	154 (72.6)	170 (80.2)	173 (81.6)	
Other Races	35	14 (6.6)	12 (5.7)	9 (4.2)	
**Ethnicity**					0.232
Hispanic or Latino	22	11 (5.2)	6 (2.8)	5 (2.4)	
Not Hispanic or Latino	614	201 (94.8)	206 (97.2)	207 (97.6)	
**Income**					0.001
< $20,000	143	60 (28.3)	47 (22.2)	36 (17.0)	
$20,000-$65,000	341	114 (53.8)	126 (59.4)	101 (47.6)	
> $65,000	152	38 (17.9)	39 (18.4)	75 (35.4)	
**Education**					0.098
<=High School Diploma	179	70 (33.0)	58 (27.4)	51 (24.1)	
Some College	253	88 (41.5)	81 (38.2)	84 (39.6)	
>=4-year Degree	204	54 (25.5)	73 (34.4)	77 (36.3)	
**Maternal Education**					0.74
<=High School Diploma	413	134 (63.2)	136 (64.2)	143 (67.5)	
Some College	102	37 (17.5)	37 (17.5)	28 (13.2)	
>=4-year Degree	121	41 (19.3)	39 (18.4)	41 (19.3)	
**House Type**					<0.001
Single Family Structure	528	145 (68.4)	183 (86.3)	200 (94.3)	
Multi-family Structure	118	67 (31.6)	29 (13.7)	12 (5.7)	
**Employment Status**					0.993
Employed	383	127 (59.9)	126 (60.4)	126 (60.4)	
Not Employed	253	85 (40.1)	84 (39.6)	84 (39.6)	
**Continuous** - mean (SD)					
**Age**		48.2 (12.8)	48.5 (12.1)	50.4 (13.3)	0.152
**Traffic Density, 500 m radius (x1000)**		66.78 (57.77)	68.68 (53.94)	52.44 (47.03)	0.003
**Area Population Density (×1000)**		2.50 (0.74)	2.53 (0.61)	2.30 (0.52)	<0.001
**NDVI 100 m Radius**		0.16 (0.04)	0.22 (0.01)	0.27 (0.02)	

**Table 2:** Adjusted associations between individual- and neighborhood-level SES indicators and greenness.

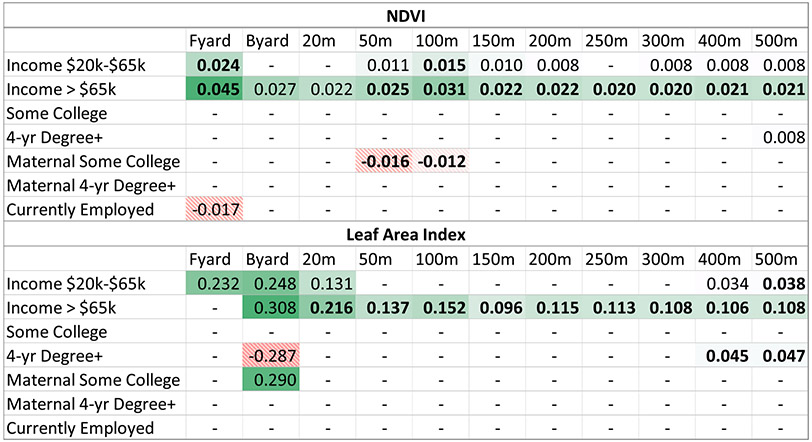

Notes: N = 636. Models adjusted for participants’ age, gender, race, income, education, mother’s education, employment status, and traffic density. Parameter coefficient estimates from linear regression model. Only coefficient estimates with p < 0.10 are shown. Coefficients estimates with p < 0.05 are shown in bold and identified in the text as significant, with p = 0.05–0.10 identified as marginally significant. Coefficient estimates with p > 0.10 are replaced with a dash (−). Table cells are colored by effect size on a dark to white scale, green solid color for positive significant or marginally significant associations and dashed red for inverse associations. Fyard = participants’ front yard, Byard = participants’ back yard. Reference groups include for Income, < $20,000; for education, ≤high school graduate; for maternal education, ≤high school graduate; employment, not currently employed.

**Table 3:** No-cost tree planting adoption by participant characteristics and neighborhood factors. Notes: Chi-sq (categorical data) and ANOVA (continuous data) results shown correlating participant and area characteristics with planting success. Study participants residing at single family residential structures located within the planting intervention area. Ethnicity category removed as only 1 participant in the planting area reported their ethnicity as Hispanic or Latino.

Categorical – n (%)	Total, N = 215	Unplanted, N = 120	Planted, N = 95	p- value
**Gender**				0.496
Female	140	81 (67.5)	59 (62.1)	
Male	75	39 (32.5)	36 (37.9)	
**Race and Etnicity**				0.393
Black	20	14 (11.7)	6 (6.3)	
White	189	103 (85.8)	87 (91.6)	
Other Races	5	3 (2.5)	2 (2.1)	
**Income**				0.173
< $20,000	35	23 (19.2)	12 (12.6)	
$20,000-$65,000	130	66 (55.0)	64 (67.4)	
> $65,000	50	31 (25.8)	19 (20.0)	
**Education**				0.130
<=High School Diploma	56	34 (28.3)	22 (23.2)	
Some College	80	49 (40.8)	31 (32.6)	
>=4-year Degree	79	37 (30.8)	42 (44.2)	
**Mother’s Education**				0.071
<=High School Diploma	138	85 (70.8)	53 (55.8)	
Some College	32	14 (11.7)	18 (18.9)	
>=4-year Degree	55	21 (17.5)	24 (25.3)	
**Employment Status**				0.934
Employed	134	74 (61.7)	60 (73.8)	
Not Employed	81	46 (38.3)	35 (36.8)	
**Continuous** - mean (SD)				
**Age**		47.6 (12.6)	48.2 (14.3)	0.757
**Lot Size (Sq.m.)**		571.8 (240.9)	643.6 (285.8)	0.047
**Front Yard NDVI**		0.23 (0.09)	0.23 (0.08)	0.669
**Back Yard NDVI**		0.28 (0.09)	0.28 (0.08)	0.687
**100 m Radius NDVI**		0.22 (0.04)	0.23 (0.08)	0.016
**500 m Radius NDVI**		0.21 (0.03)	0.22 (0.03)	0.07
**Front Yard LAI**		1.32 (1.37)	1.13 (1.18)	0.297
**Back Yard LAI**		1.49 (1.21)	1.65 (1.20)	0.375
**100 m Radius LAI**		1.08 (0.25)	1.20 (0.29)	0.001
**500 m Radius LAI**		1.02 (0.15)	1.08 (0.14)	0.003
**Assessed Home Value (×1000)**		83.2 (40.4)	97.6 (46.9)	0.016
**Traffic Density, 500 m Radius (×1000)**		66.6 (51.2)	78.2 (52.8)	0.106
**Area Population Density (×1000)**		2.7 (0.6)	2.5 (0.5)	0.01

## Data Availability

The authors do not have permission to share data.
